# Reconstruction of Extensive Calvarial Exposure After Major Burn Injury in 2 Stages Using a Biodegradable Polyurethane Matrix

**Published:** 2016-05-09

**Authors:** John Edward Greenwood, Marcus James Dermot Wagstaff, Michael Rooke, Yugesh Caplash

**Affiliations:** ^a^Adult Burn Service, Royal Adelaide Hospital, Adelaide, South Australia; ^b^Department of Plastic and Reconstructive Surgery, Royal Adelaide Hospital, Adelaide, South Australia

**Keywords:** synthetic, biodegradable polyurethane, dermal matrix, scalp reconstruction, burns

## Abstract

**Objectives:** To share our experience of an extensive calvarial reconstruction in a severely burn-injured, elderly patient in a 2-stage procedure utilizing a novel biodegradable temporizing matrix (NovoSorb BTM), followed by autograft. **Materials and Methods:** A 66-year-old patient with 75% full-thickness burns, including 7% total body surface area head and neck, with calvarial exposure of approximately 350 cm^2^, complicated by acute renal failure and smoke inhalation injury. Exposed calvarium was burred down to diploe and biodegradable temporizing matrix was applied. Over the next 29 days, the biodegradable temporizing matrix integrated by vascular and tissue ingrowth from the diploe. Delamination and grafting occurred, however, at 43 days postimplantation of biodegradable temporizing matrix due to skin graft donor-site constraints. **Results:** Graft take was complete, yielding a robust and aesthetically pleasing early result (26 days post–graft application). **Conclusions:** Biodegradable temporizing matrix offers an additional resource for reconstructive surgeons faced with fragile patients and complex wounds.

Soft-tissue coverage of calvarial exposure represents a significant surgical challenge. The technique employed is determined by several factors, and an excellent algorithmic approach was published recently.^1^ The common causes of calvarial exposure reported include trauma, tumor extirpation (skin cancers or intracranial lesions, with or without secondary radiotherapy), osteoradionecrosis, chronic osteomyelitis, and electrical injury.^2^^,^^3^ Options open to the reconstructive surgeon for calvarial coverage are relatively broad because the defects caused are usually relatively small,^2^^,^^4^^-^8 although larger defects occasionally result.^9^ Many approaches are impractical in acutely burn-injured patients, who need to have their wounds controlled quickly but who lack both the physiological reserve to tolerate long procedures and the unburned resources to allow free tissue transfer.

Dermal substitutes have found value and popularity in reconstruction of the exposed calvarium,^4^^-^9 although more commonly in post–cancer treatment. By burring the outer table and placing the product on to the diploe, a potentially contaminated wound bed can be converted to a clean vascularized structure safely and quickly by an experienced surgeon. Exposed calvarium can be physiologically “closed” early on in the patient's treatment, with minimal systemic disruption. This allows treatment efforts to be directed toward optimizing patient comorbidities and recovery, enabling them later to tolerate more insulting secondary reconstructive surgery and allowing previously harvested graft donor sites to heal robustly.

There are limitations with commercially available biological dermal substitutes, particularly with regard to cost^10^ and risk of infection.^11^^,^^12^

We describe a 66-year-old male patient with 75% total body surface area (TBSA) full-thickness burns, inhalation injury, and acute renal failure, who was colonized with several pathological microbes, both bacterial and fungal. The burns included 7% TBSA full-thickness injury to the head and circumferential neck, sparing only the eyes and midface with calvarial exposure measuring approximately 18 × 19 cm (∼350 cm^2^).

## CASE PRESENTATION

A rapidly moving bushfire passing across 85,000 hectares (210,000 acres) destroyed a small town, claimed 2 lives and injured 13. Five of those injured sustained significant burns and were admitted to the Adult Burns Service at the Royal Adelaide Hospital. The most significant burn injury, 75% TBSA full-thickness burns, in a 66-year-old male patient was complicated by smoke inhalation injury and renal failure. The burns included the head and neck, anterior and posterior trunk, both arms, and both lower legs. The feet, buttocks, perineum, parts of both thighs, midface, and left axilla were spared.

The patient arrived to the emergency department and was intubated and ventilated. He underwent escharotomies to all limbs and trunk during his trauma clearance, before attending the operating theatre. Within 6 hours of his injury, his burns to both upper limbs had been excised to fascia, revealing death of the right distal forearm muscle compartments both on the volar side and dorsally and at-risk viability of the digits of the left hand. Deep burns of the chest and abdomen were also excised to fascia as were circumferential burns of both lower limbs. Although head burns were obvious, these were cleaned and left at this first operation and, due to falling core temperature and physiological lability, deep burns on his back also had to be left. His wounds were dressed, and he was admitted on to the intensive care unit (ICU).

Two days later, he returned to operating theatre where a below-elbow amputation was performed on the right. The remaining deep burn was removed from the posterior trunk and, under the Australian Therapeutic Goods Administration (TGA) Special Access Scheme, a biodegradable polyurethane dermal matrix (NovoSorb BTM; PolyNovo Biomaterials Pty Ltd, Port Melbourne, Victoria, Australia) was implanted into all debrided burn wounds (anterior and posterior trunk, both arms and legs). This concluded the majority of his early burn surgery until the BTM was integrated and ready to graft at day 40 postimplantation.

**Figure 1:**
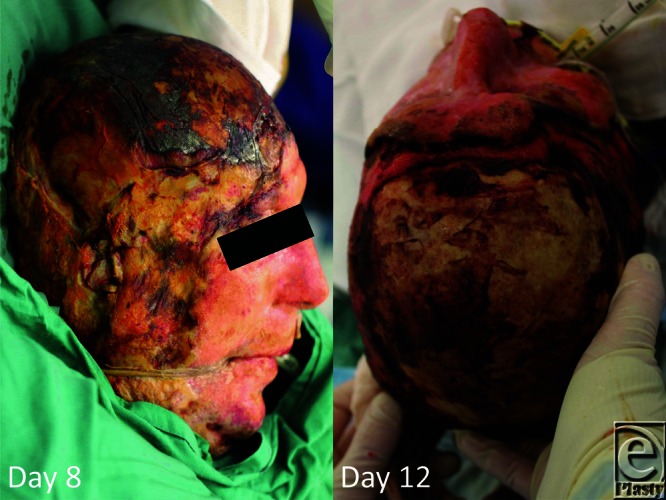
Scalp burn degradation necessitating excision at day 6, progressing to reveal extensively exposed calvarium by day 12.

**Figure 2:**
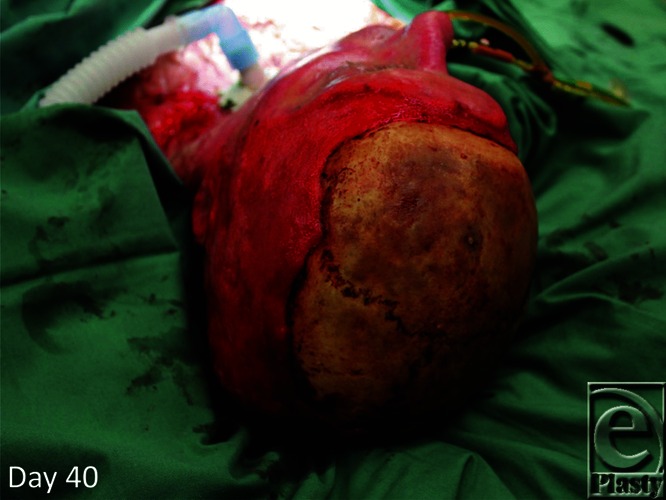
Slow and meticulous debridement of the head and neck burns resulted in a clean, granulating head and neck. Full calvarial exposure demonstrated.

On day 6 postinjury, however, his head and neck burns began to declare and degrade, necessitating excision of the scalp and dead periosteum to the level of the skull over the entire calvarium (Fig 1). It took until day 40, with meticulous small debridements designed to retain as much viable head tissue as possible before the wounds were clean around the exposed calvarium (Fig 2). In the intervening period, further necrosis was evident on the right upper limb, revealing an open elbow joint, and the amputation was converted to above-elbow. In addition, the BTM on both lower limbs and left upper limb had failed to integrate and been removed, the underlying wounds cleaned, and negative pressure wound therapy (VAC) dressings applied.

At this stage, he was still resident on the ICU with a tracheostomy in situ. The BTM over the chest, abdomen, back, and residual right upper limb had completely integrated. The lower limb and left upper limb wounds were clean and lightly granulating. Thus, on day 43, he underwent his third major surgery. Fresh BTMs were implanted into the lower limbs. The integrated BTMs were delaminated (the outer seal peeled off) and the neodermis refreshed by light dermabrasion with a diamond-tipped cylindrical burr. Meshed split skin autograft (1:3) was applied to the integrated BTM over the chest, abdomen, back, right arm stump, and both shoulders as well as into the left arm wounds (where BTM had previously failed). Finally, our attention turned to the head.

## MATERIALS AND METHODS

Figure 2 shows the extent of the calvarial exposure once serial head burn debridement was complete. The periosteum of the calvarium had been lost and the health of the exposed outer table was in doubt. Options for reconstruction were limited and the physiological reserve of the patient insufficient to attempt major surgery. A computed tomographic scan performed after admission was retrospectively reviewed to confirm the presence of a diploic space between the inner and outer calvarial tables.

Using a dental “olive” burr, a craniofacial colleague removed the outer skull table to expose the underlying diploe (see Videos 1 and 2, available at: …). Perforating bleeding vessels were occluded with a small amount of bone wax. The outer table removal extended up to the margin of granulation, which had formed on the surrounding viable tissue.

**Figure 3:**
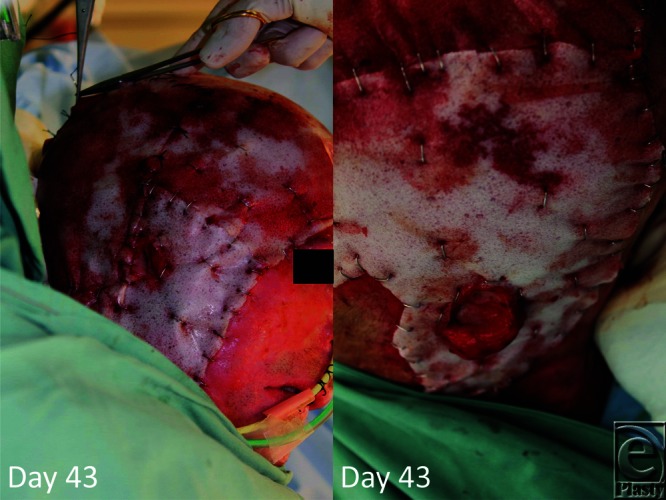
Following burring of the outer table, BTM was applied like a “bathing cap.” BTM indicates biodegradable temporizing matrix.

BTM was applied over the whole wound (granulations and diploe) and cut to shape with scissors. “Darts” were needed to accommodate the curved surface. Where the BTM overlay granulation, it could be held with staples. However, seams over the diploe were held together with sutures. The final result resembled a “scrum cap” (Fig 3). Overdressing was with Acticoat (again darted to allow for the curvature of the surface) and a 4-in crêpe head bandage.

**Figure 4:**
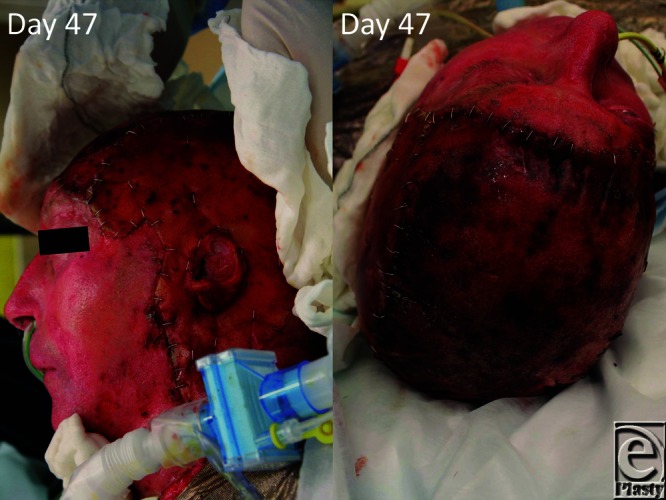
Only 4 days later, the polyurethane foam structure is still visible through the BTM seal but the pores are filled with cherry-red fluid. Less foam structure is visible laterally where BTM overlies granulating soft tissue. BTM indicates biodegradable temporizing matrix.

A first look at 4 days revealed an appearance very similar to BTM applied to other wounds. The foam structure over the calvarium was visible, but the matrix was filled with cherry-red fluid. Subsequent reviews demonstrated disappearance of the foam structure as integration by tissue progressed (Fig 4). The skin graft was taking well over the trunk and arms.

**Figure 5:**
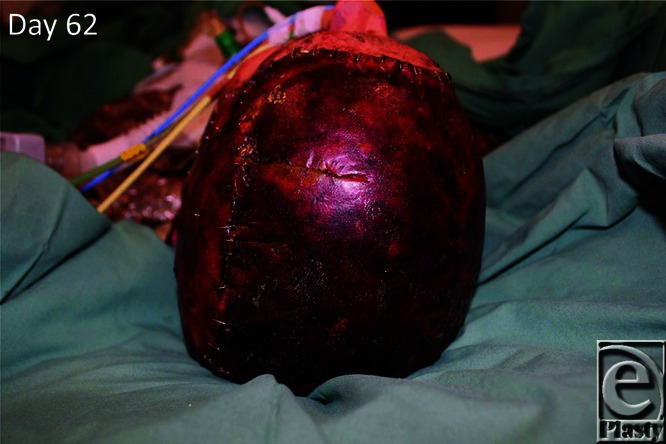
By day 62 (day 19 postimplantation), the BTM is ready for delamination. BTM indicates biodegradable temporizing matrix.

Although by 29 days after BTM application to the calvarium, the material appeared clinically ready to be delaminated and take graft (Fig 5), the previously harvested donor sites had not robustly epithelialized. It was, therefore, 46 days post-BTM application that delamination and grafting occurred.

**Figure 6:**
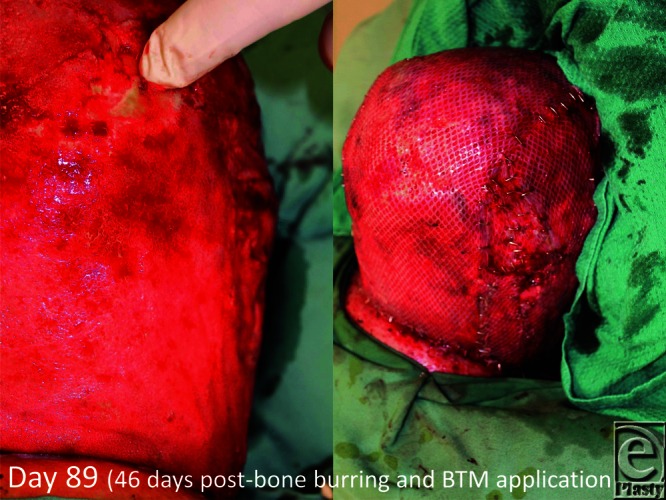
On day 89 (day 46 post-BTM application), the material seal is removed (delamination) and meshed split skin graft applied. BTM indicates biodegradable temporizing matrix.

There were no issues with delamination of the BTM sections. Each seal was peeled off in 1 piece and in 1 action. A boggy area, over the right occiput (which we believed was a “pressure” area) was revealed by delamination (Fig 6). Surface refreshment of the whole delaminated BTM (including this area) was lightly performed by dermabrasion with a diamond-tipped cylindrical burr and 1:10,000 adrenaline-soaked packs applied for rapid hemostasis. The harvested autograft was meshed at 1:1.5 to give the best cosmetic result on the head (although BTM elsewhere, including both legs at this sitting, received 1:3 mesh). The grafts were stapled into place (Fig 6) and overdressed with paraffin gauze, betadine-soaked Nufold gauze, and a crêpe head bandage.

## RESULTS

**Figure 7:**
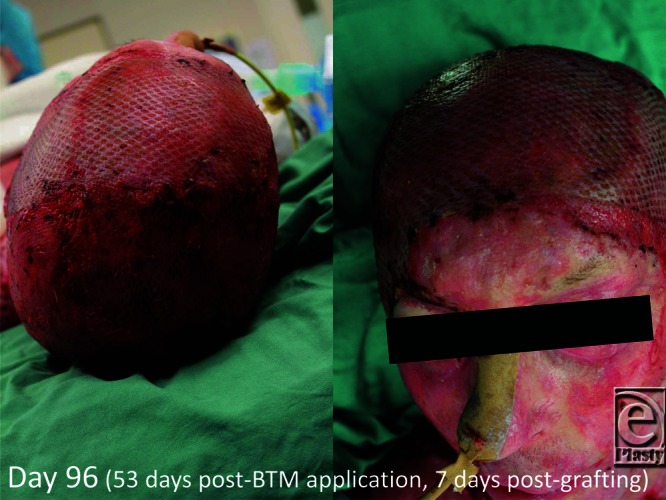
First graft look 4 days later.

**Figure 8:**
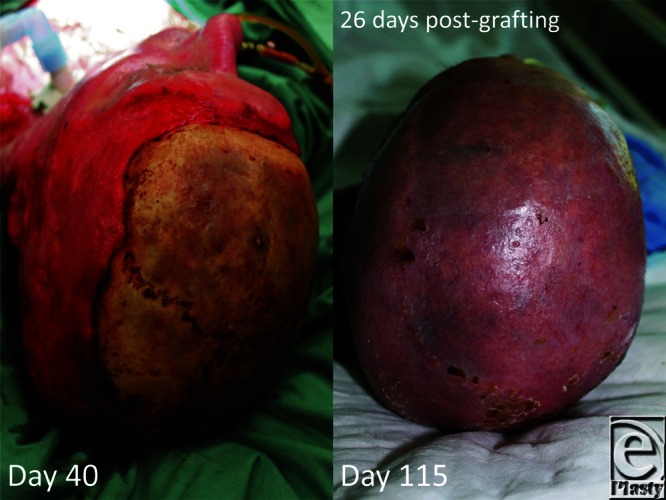
Graft appearance over the reconstructed scalp 26 days postapplication.

The dressing was refreshed 2 days later to reveal early and robust graft take. At 7 days postgrafting, the results were very good (Fig 7). The legs, which had also been grafted on the BTM that had been replaced, also sustained good skin graft take. None of these areas necessitated further revision split skin grafting. By 11 days postgrafting, the result was already robust, and Figure 8 demonstrates the comparison of this result with prereconstruction. We will keep the patient under close review for any potential mid–long-term complications of this technique, such as calvarial osteomyelitis, as part of his continued care.

In summary, his sequence of surgery is as follows:

1. Day 0: Debridement of arms, legs, anterior trunk, posterior trunk, and temporizing application of Biobrane dressing.
2. Day 2: Removal of Biobrane dressing, below-elbow amputation of the right arm. Debridement of lower posterior trunk. Application of BTM to both arms, legs, and anterior and posterior trunk.
3. Interval dressing changes in the operating theatre twice a week with serial debridement of scalp to dry calvarium. BTM on legs and left arm was removed because of failure to integrate and VAC dressings were applied.
4. Day 43: Above-elbow amputation of the right arm. Delamination, dermabrasion of BTM surface, and split skin graft to both arms and anterior and posterior trunk. Burring of calvarial outer table and application of BTM. Repeat application of BTM to legs.
5. Day 89 (once donor sites had reepithelialized): Delamination of scalp BTM, dermabrasion of BTM surface, and application of 1:1.5 meshed split skin graft to scalp and 1:3 meshed split skin graft to BTM on legs.

## DISCUSSION

This patient posed some very difficult questions, which required aggressive surgical answers. In the absence of his physiological lability and very extensive TBSA full-thickness burn, a standard algorithm such as that offered in the literature might have been applied. With regard to the unique extent of area of exposed calvarium, one approach we would have otherwise considered was the use of a latissimus dorsi free flap and autograft coverage in 1 stage. This would have been a long and hazardous procedure, compounded by his extensive back burns over the flap donor access area, and deep wounds over both sides of the neck and preauricular regions where microsurgery would be performed. In the absence of multiresistant *Pseudomonas*, *Stenotrophomonas*, and *Scedomycosis* (*Scedosporium apiospermum*), the calvarial reconstruction might have been performed with a collagen-based dermal matrix, but the risk of infection was deemed too great.

Our department has gained significant experience in the use of BTM throughout its development from animal studies to 3 human clinical trials, the most recent of which was its use in 5 patients suffering from 20% to 50% TBSA full-thickness or deep dermal burns, which has recently completed recruitment and acute surgery and the last patient has been discharged from acute care. Although unapproved, and not commercially available, we were able to use it for patients of the bushfire via the TGA Special Access Scheme. The BTM offered a 2-stage reconstruction—the first stage using an inexpensive material, which has previously demonstrated to us robustness in the face of infection, physiologically covering the exposed bone while *not* attempting too early reharvest of graft resources. The second stage involved BTM delamination, neodermis dermabrasion, autograft harvest, and application. Both stages were short, reducing anesthetic insult, and the potential for in-theatre core temperature loss.

## CONCLUSIONS

This novel, biodegradable, polyurethane-based material offered a hitherto unavailable resource and safely allowed us to exploit a previously described technique for calvarial coverage and reconstruction. The process minimized physiological insult to the patient during critical early treatment and allowed us to stage the reconstruction to best optimize robustness of the skin graft donor sites for reharvest. In the general literature, burns exposing the calvarium are usually the sequelae of major, normally fatal, burn injuries, with patients not surviving to a point where reconstruction can be considered. However, BTM represents a new, effective, and rapid solution for temporizing such burns, which might mean that we see more of these cases surviving in future.
